# Supplementary material for: Biosynthetic gene cluster profiling predicts the positive association between antagonism and phylogeny in Bacillus
Source: Nat Commun. 2022 Feb 23;13:1023. doi: 10.1038/s41467-022-28668-z (PMC8866423; doi:10.1038/s41467-022-28668-z)
Supplement: Supplementary file 1 — Supplementary Information [file 41467_2022_28668_MOESM1_ESM.pdf]

Supplementary Information for

Biosynthetic gene cluster profiling predicts the positive association between antagonism and phylogeny in *Bacillus*

The following file includes:

Supplementary Figures 1 to 9

Supplementary Table 1

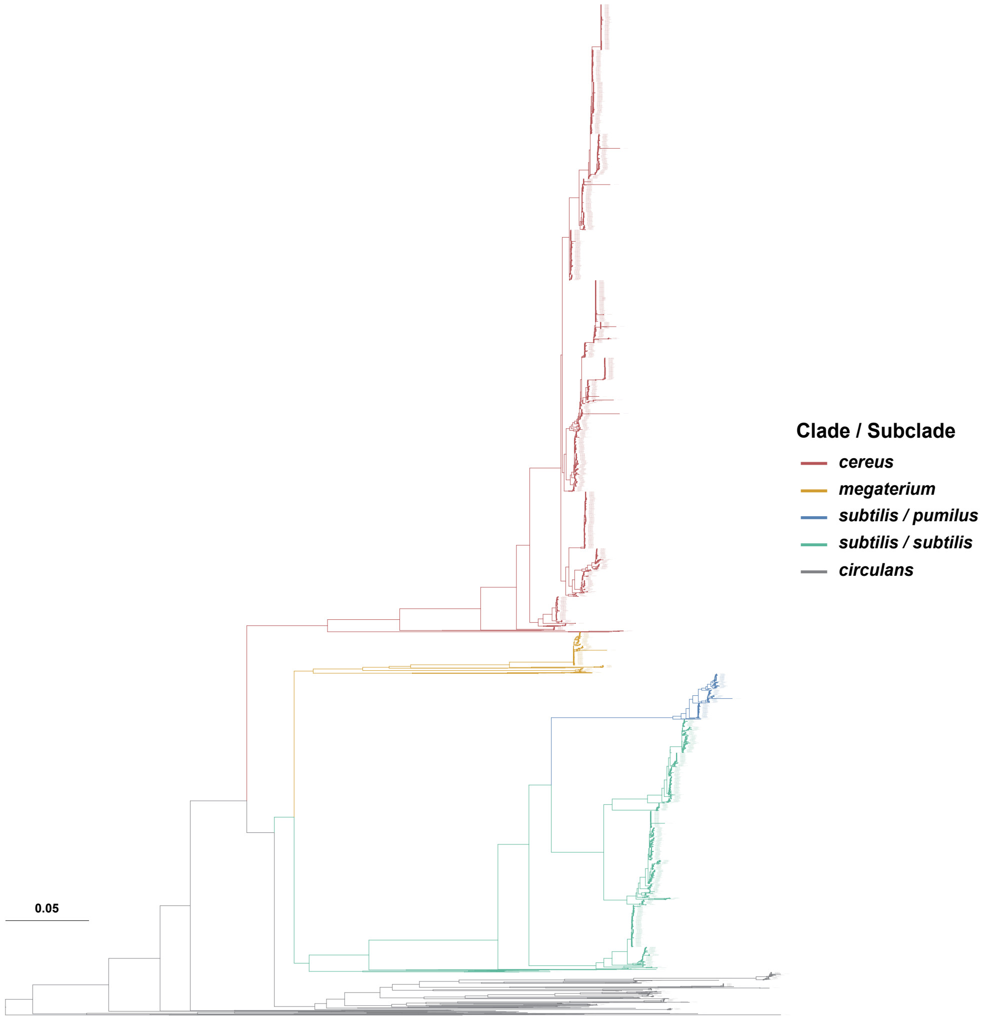

Supplementary Figure 1. Maximum likelihood (ML) phylogenetic tree of the 4,268 *Bacillus* genomes based on the sequences of 120 ubiquitous single-copy proteins[27]. This phylogram is the fully-annotated version of Fig. 1, which shows the detailed species information of the *Bacillus* genomes.

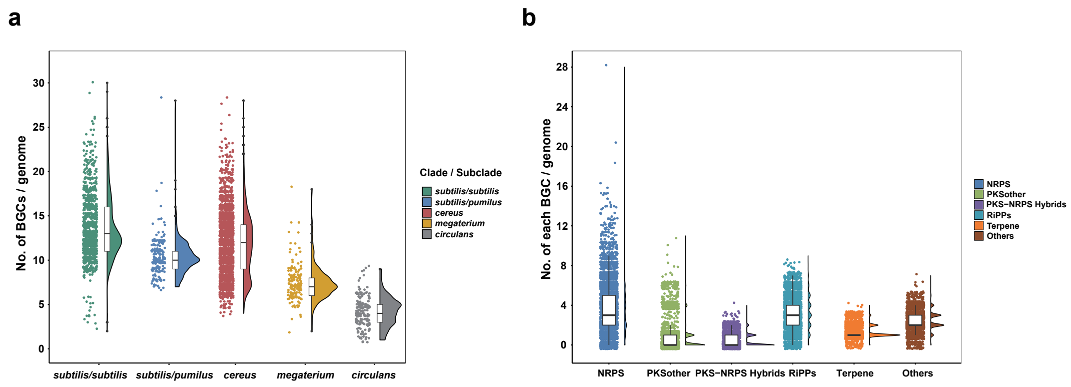

Supplementary Figure 2. Statistics of BGCs belonging to the 4,268 *Bacillus* genomes. a Statistics of BGCs from different *Bacillus* (sub)clades. **b** Statistics of BGCs from different BiG-SCAPE classes. NRPS, nonribosomal peptide-synthetase; PKS, polyketide synthase; RiPPs, ribosomally synthesized and post-translationally modified peptides. BGCs belonging to polyketide synthase type I (PKSI) or extracellular polysaccharides (EPS) are not shown since they are extremely rare in *Bacillus* genomes. In the violin plot, the centre line represents the median, violin edges show the 25th and 75th percentiles, and whiskers extend to 1.5× the interquartile range.

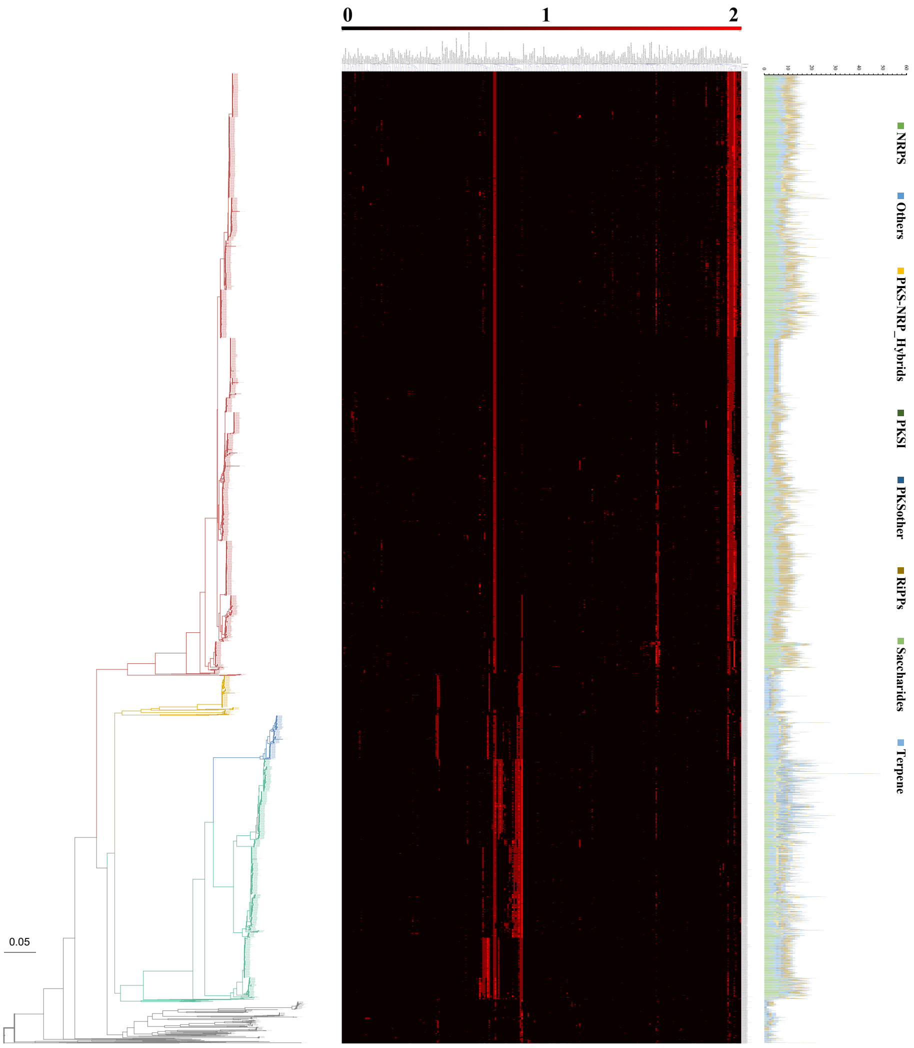

Supplementary Figure 3. Profile of BGC products and classification attached to the phylogenetic tree of total 4,268 *Bacillus* genomes. Matching to the genome order in the phylogenetic tree (the same as that in Fig. 1 & Supplementary Figure 1), BGCs profile in each genome were shown as the number of 256 detailed product types (Supplementary Data 3) by the heatmap (which were further clustered only at the level of BGC products), as well as the number of 8 different classifications (NPRS, PKSI, PKSother, PKS-NRP_Hybrids, RiPPs, Saccharides, Terpene, and Others) through the histogram.

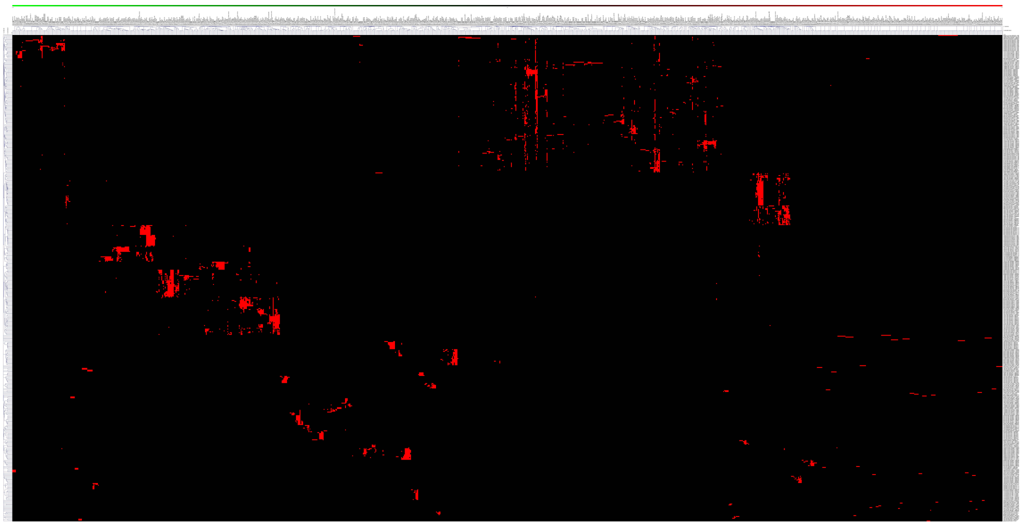

Supplementary Figure 4. Hierarchal clustering among the 545 representative *Bacillus* genomes based on the abundance of the different biosynthesis gene cluster families (GCFs). This figure is the fully-annotated version of Fig. 2b. Each row represents a GCF and has been noted with the potential BGC product, which was classified through BiG-SCAPE by calculating the Jaccard index (JI), adjacency index (AI), and domain sequence similarity (DSS) of each BGC[28]. Each line represents a *Bacillus* genome, and the abundance of each GCF in different genomes is shown in the heatmap. The left tree was constructed based on the distribution pattern of BGCs from different families, which showed a similar pattern to the phylogram in Fig. 1.

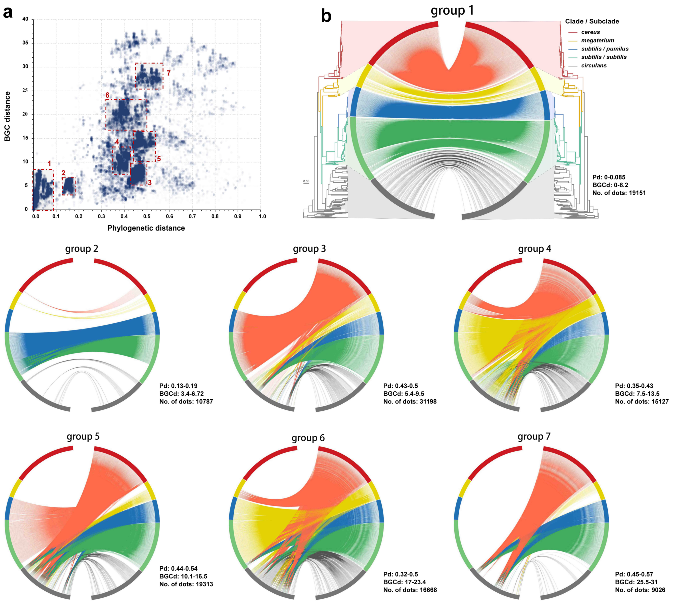

Supplementary Figure 5. Connection of *Bacillus* genomes in 7 groups showing different correlation between BGC distance and phylogenetic distance. a The correlation between BGC distance and phylogenetic distance of the 545 representative *Bacillus* genomes was showed as a dot plot, in which 7 high-density distinguishable dot areas forms (group 1~7, with 19151 (12.6%), 10787 (7.1%), 31198 (20.4%), 15127 (9.9%), 19313 (12.7%), 16668 (10.9%), and 9026 (5.9%) dots, respectively). **b** For detail analysis, the points of the 7 groups in (a), corresponding to the relationships of each two different *Bacillus* genomes, were extracted, respectively, and shown as the line directly on the phylogenetic tree of the 545 *Bacillus* genomes. Both semicircles consists of the 545 *Bacillus* genomes with the same order as in the phylogenetic tree (e.g., group 1 in (b)); each line indicates that the BGC distance and phylogenetic distance of the connected pairwise genomes, represent a dot in the corresponding area/group in (a). Different colors were used to indicate each *Bacillus* clades/subclades: red, *cereus* clade; blue, *pumilus* subclade; green, *subtilis* subclade; yellow, *megaterium* clade; gray, *circulans* clade. Pd: phylogenetic distance; BGCd: BGC distance. Source data are provided as a Source Data file.

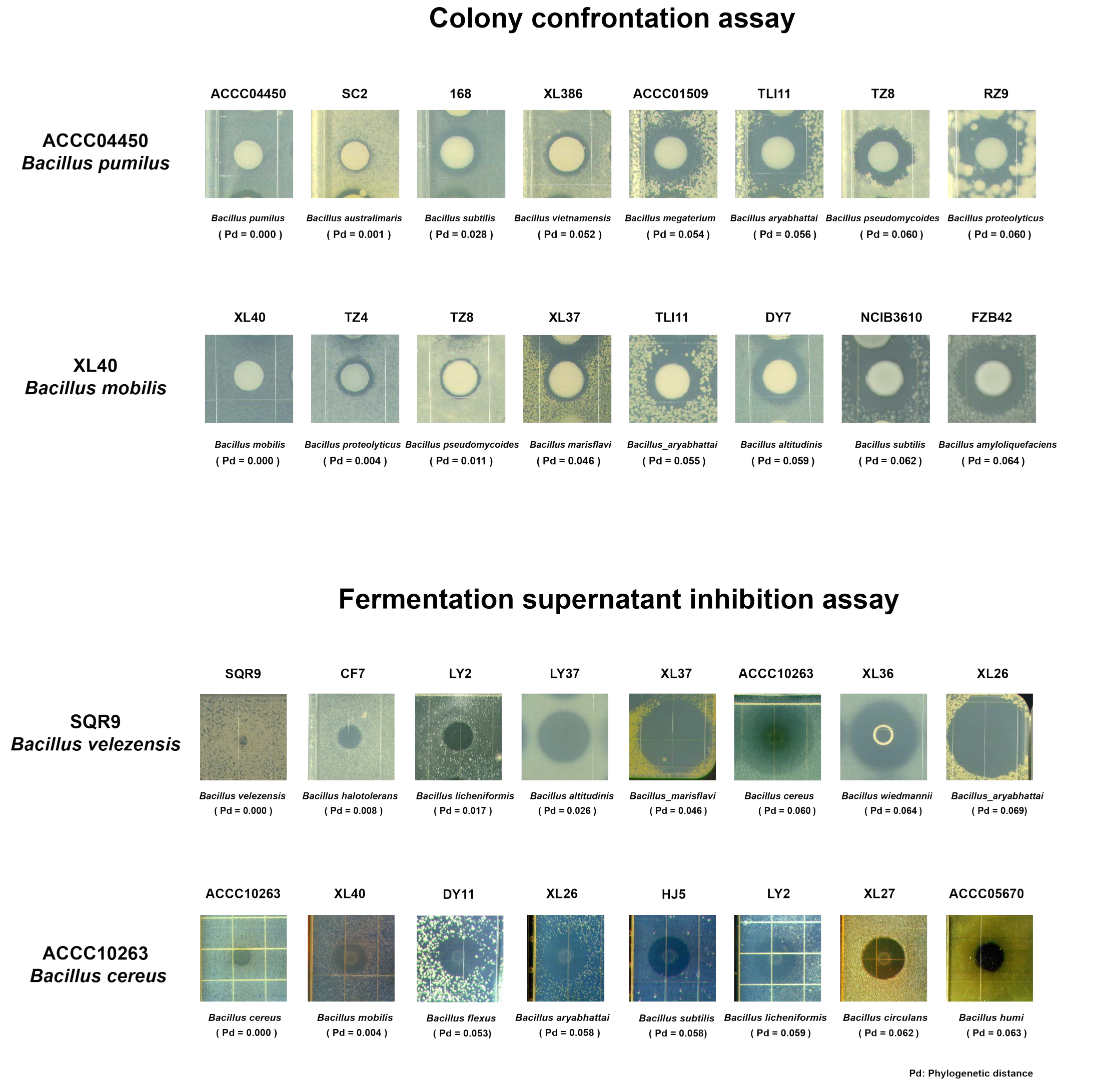

Supplementary Figure 6. Visual representations of colony confrontation and fermentation supernatant inhibition assay.

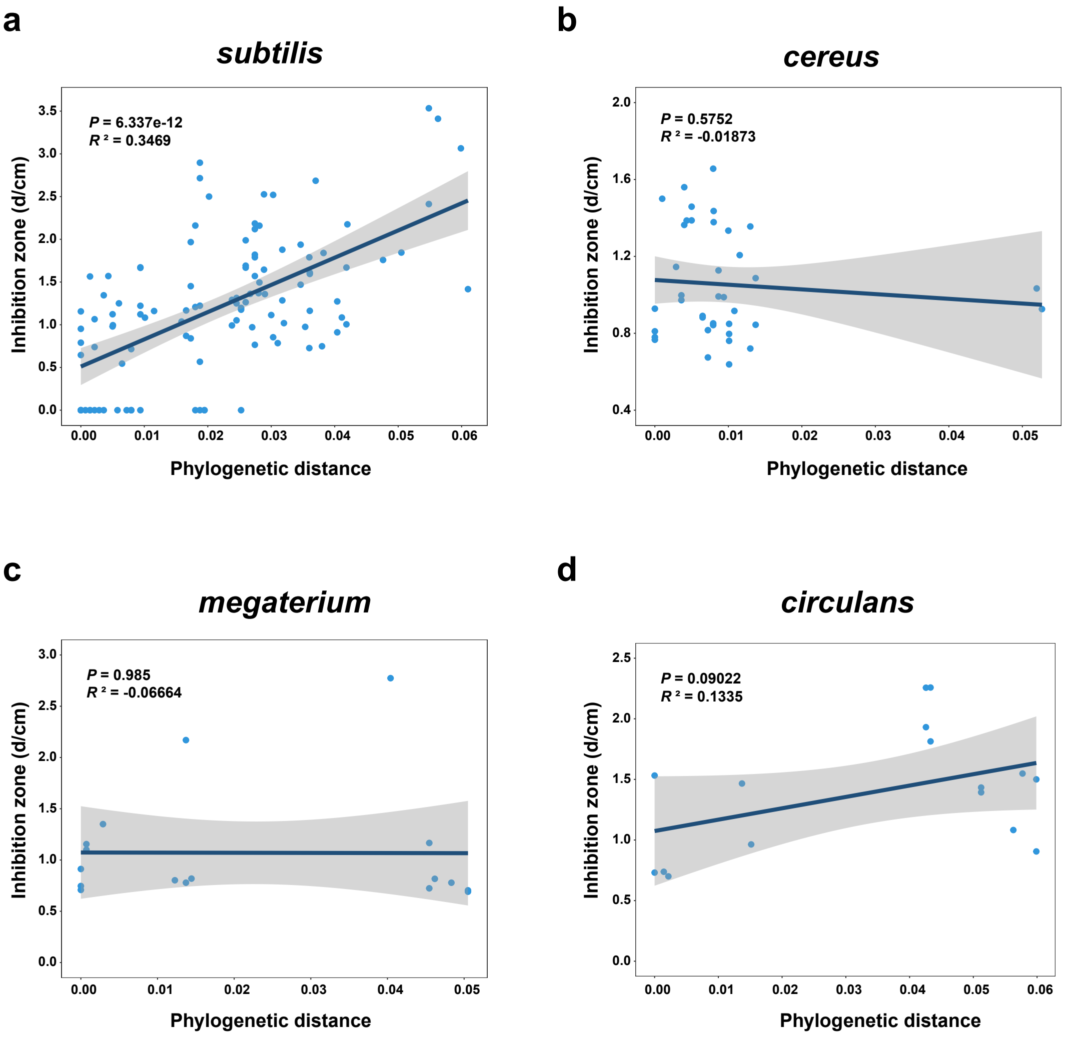

Supplementary Figure 7. Correlation between the antagonism phenotype (diameter of the inhibition zone in the fermentation supernatant assessment) and 16S rDNA phylogenetic distance among bacterial strains within the four *Bacillus* clades. a Correlation within the *subtilis* clade, F-statistic: 59.43 on 1 and 109 DF, $P = 6.337 \times 10^{-12}$, $R^2 = 0.3469$; **b** Correlation within the *cereus* clade, F-statistic: 0.3198 on 1 and 36 DF, $P = 0.5752$, $R^2 = -0.01873$; **c** Correlation within the *megaterium* clade, F-statistic: 0.0003673 on 1 and 15 DF, $P = 0.985$, $R^2 = -0.06664$; **d** Correlation within the *circulans* clade, F-statistic: 3.312 on 1 and 14 DF, $P = 0.09022$, $R^2 = 0.1335$. The error bands indicate the 95% confidence intervals. Linear model (LM) was used for the correlation analysis and adjustments were made for R^2 calculation; one-sided F test was applied for multiple comparisons. Source data are provided as a Source Data file.

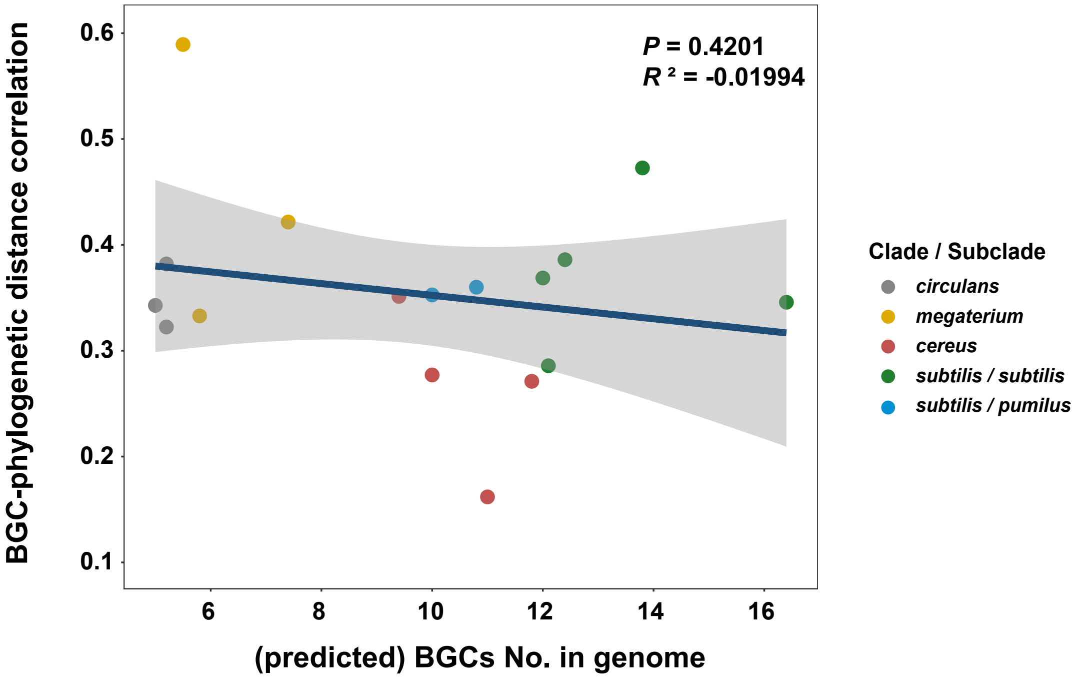

Supplementary Figure 8. Correlation of the BGC-phylogenetic distance association with the (predicted) quantity of BGCs in antagonistic strains. For strains whose genomes have not been completely sequenced, we referred to the average quantity of BGCs in this species (Supplementary Data 8). The color of the dots represents the clade/subclade which the antagonistic strains belong to. The error bands indicate the 95% confidence intervals. Linear model (LM) was used for the correlation analysis and adjustments were made for R^2 calculation (P = 0.4201, R^2 = -0.01994); one-sided F test was applied for multiple comparisons (F-statistic: 0.6872 on 1 and 15 DF). Source data are provided as a Source Data file.

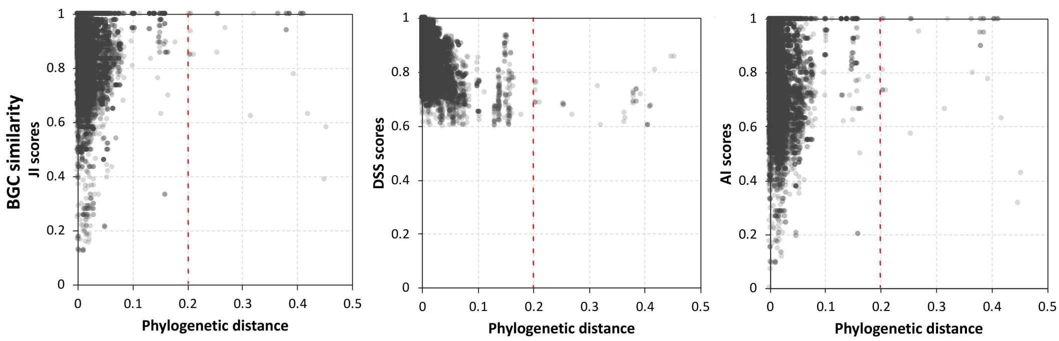

Supplementary Figure 9. Correlation between BGC similarity and phylogenetic distance in all distinct BGC families from the 545 representative *Bacillus* genomes. BGCs in the same BGC family identified by BiG-SCAPE software were considered as homologous, and thus are suitable for horizontal gene transfer (HGT) analysis in different *Bacillus* species. For detail, pairwise distance between BGCs within each family was calculated by combining three similarity scores from Jaccard Index (JI), Adjacency Index (AI), and Domain sequence similarity (DSS) analyses, and then matched to the phylogenetic distance of their derived *Bacillus* genomes. For each analysis (JI, DSS, or AI), the correlations from all BGC families were merged together, and those points with phylogenetic distance ≥ 0.2 (indicating the pairwise genomes are from different clades or at least distant species within clade) and similarity scores ≥ 0.4 (indicating the pairwise genomes accommodate homologous BGCs) were possible consequence from HGT events. Source data are provided as a Source Data file.

Supplementary Table 1. Statistics of different classes of biosynthetic gene clusters (BGCs) in different *Bacillus* clades.

Clade	No. of genomes	No. of BGCs belong to different classifications																	
		NRPS		PKSother		PKSI		PKS-NRPS Hybrids		RiPPs		Saccharides		Terpene		Others		Total	
		No.	Average	No.	Average	No.	Average	No.	Average	No.	Average	No.	Average	No.	Average	No.	Average	No.	Average
subtilis	1259	4661	3.7	2508	2.0	4	0.0	922	0.7	2512	2.0	0	0.0	2050	1.6	3845	3.1	16502	13.1
cereus	2652	11168	4.2	47	0.0	10	0.0	561	0.2	10433	3.9	1	0.0	2654	1.0	6199	2.3	31073	11.7
megaterium	176	41	0.2	191	1.1	0	0.0	11	0.1	257	1.5	0	0.0	485	2.8	326	1.9	1311	7.4
circulans	181	55	0.3	150	0.8	0	0.0	2	0.0	195	1.1	0	0.0	225	1.2	158	0.9	785	4.3
**Total**	4268	15925	3.7	2896	0.7	14	0.0	1496	0.4	13397	3.1	1	0.0	5414	1.3	10528	2.5	49671	11.6
