# Supplementary material for: Biosynthetic gene cluster profiling predicts the positive association between antagonism and phylogeny in Bacillus
Source: Nat Commun. 2022 Feb 23;13:1023. doi: 10.1038/s41467-022-28668-z (PMC8866423; doi:10.1038/s41467-022-28668-z)
Supplement: Supplementary file 3 — Description of Additional Supplementary Files [file 41467_2022_28668_MOESM3_ESM.docx]

Description of Additional Supplementary Files

Title: Supplementary Data 1.

Description: Information of the 4,268 Bacillus genomes used for phylogenetic analysis and biosynthetic gene clusters (BGCs) prediction.

Title: Supplementary Data 2.

Description: Taxonomic distribution of genomes across different species and clades.

Title: Supplementary Data 3.

Description: Prediction of biosynthetic gene clusters (BGCs) in all 4,268 Bacillus genomes by using antiSMASH.

Title: Supplementary Data 4.

Description: Biosynthesis gene cluster families (GCFs) and gene cluster clans (GCCs) in the representative 545 Bacillus genomes based on interactive sequence similarity network analysis using BiG-SCAPE.

Title: Supplementary Data 5.

Description: Information of each predicted biosynthesis gene cluster family (GCF) and gene cluster clan (GCC) in the representative 545 Bacillus genomes.

Title: Supplementary Data 6.

Description: Statistics of biosynthetic gene cluster families (GCFs) in the 545 representative Bacillus genomes. The order of the genomes in column and GCFs in line is consistent with that in the Heatmap of Fig. 2b & Supplementary Figure 4.

Title: Supplementary Data 7.

Description: Bacterial strains used in this study.

Title: Supplementary Data 8.

Description: Quantity of specific biosynthetic gene clusters (BGCs) of each antagonistic strain when confronted with different target strains in the fermentation supernatant inhibition assay. For strains whose genome have not been completely sequenced, the BGC presence was assigned if more than 80% of the corresponding Bacillus species genomes possessed this cluster, and their total predicted BGCs No. was calculated as the average BGCs. No. in all genomes of this species.

Title: Supplementary Data 9.

Description: Primers used for mutants construction and verification.
