# Supplementary material for: Biosynthetic gene cluster profiling predicts the positive association between antagonism and phylogeny in Bacillus
Source: Nat Commun. 2022 Feb 23;13:1023. doi: 10.1038/s41467-022-28668-z (PMC8866423; doi:10.1038/s41467-022-28668-z)
Supplement: Supplementary file 13 — Reporting Summary [file 41467_2022_28668_MOESM13_ESM.pdf]

nature portfolio

Corresponding author(s): Nan Zhang & Ruifu Zhang

Last updated by author(s): Jan 11, 2022

Reporting Summary

Nature Portfolio wishes to improve the reproducibility of the work that we publish. This form provides structure for consistency and transparency in reporting. For further information on Nature Portfolio policies, see our Editorial Policies and the Editorial Policy Checklist.

Statistics

For all statistical analyses, confirm that the following items are present in the figure legend, table legend, main text, or Methods section.

n/a	Confirmed	
☐	☒	The exact sample size (*n*) for each experimental group/condition, given as a discrete number and unit of measurement
☐	☒	A statement on whether measurements were taken from distinct samples or whether the same sample was measured repeatedly
☐	☒	The statistical test(s) used AND whether they are one- or two-sided<br>*Only common tests should be described solely by name; describe more complex techniques in the Methods section.*
☒	☐	A description of all covariates tested
☒	☐	A description of any assumptions or corrections, such as tests of normality and adjustment for multiple comparisons
☐	☒	A full description of the statistical parameters including central tendency (e.g. means) or other basic estimates (e.g. regression coefficient) AND variation (e.g. standard deviation) or associated estimates of uncertainty (e.g. confidence intervals)
☐	☒	For null hypothesis testing, the test statistic (e.g. *F*, *t*, *r*) with confidence intervals, effect sizes, degrees of freedom and *P* value noted<br>*Give P values as exact values whenever suitable.*
☒	☐	For Bayesian analysis, information on the choice of priors and Markov chain Monte Carlo settings
☒	☐	For hierarchical and complex designs, identification of the appropriate level for tests and full reporting of outcomes
☒	☐	Estimates of effect sizes (e.g. Cohen's *d*, Pearson's *r*), indicating how they were calculated

Our web collection on statistics for biologists contains articles on many of the points above.

Software and code

Policy information about availability of computer code

Data collection	In total, 4,268 available genomes from 139 different Bacillus species were downloaded from the NCBI database using the ncbi-genome-download script (https://github.com/kblin/ncbigenome-download/).
Data analysis	For Bacillus genomic, phylogenetic, and biosynthetic gene cluster (BGC) analysis, we used GTDB-Tk 1.4.1, Figtree 1.4.4, MEGA 5.0, antiSMASH 5.0, BiG-SCAPE, and TIGR multiexperiment viewer (MeV 4.8.1, http://www.tigr.org/software); for visualization of the connection between Bacillus genomes, we used CIRCOS software (www.circos.ca); for Bacillus strains' 16S rRNA gene sequencing and analysis, we used Sanger dideoxy sequencing, EzBioCloud database, NCBI BLAST, and MEGA 5.0; for linear models (LM) analysis, we used R package (version 3.6.1); for heatmap drawing, we used GraphPad Prism 8.3.0; for statistical analysis of differences among treatments, we used SPSS version 22.0 (IBM, Chicago, IL, version 22.0).

For manuscripts utilizing custom algorithms or software that are central to the research but not yet described in published literature, software must be made available to editors and reviewers. We strongly encourage code deposition in a community repository (e.g. GitHub). See the Nature Portfolio guidelines for submitting code & software for further information.

Data

Policy information about availability of data

All manuscripts must include a data availability statement. This statement should provide the following information, where applicable:

- Accession codes, unique identifiers, or web links for publicly available datasets
- A description of any restrictions on data availability
- For clinical datasets or third party data, please ensure that the statement adheres to our policy

The DNA sequences from all incubation samples are deposited in the NCBI Sequence GenBank database with accession number listed in Supplementary Table S8.

March 2021

The authors confirm that the data supporting the findings of this study are available within the article or from the corresponding authors upon request.

Field-specific reporting

Please select the one below that is the best fit for your research. If you are not sure, read the appropriate sections before making your selection.

☒ Life sciences ☐ Behavioural & social sciences ☐ Ecological, evolutionary & environmental sciences

For a reference copy of the document with all sections, see nature.com/documents/nr-reporting-summary-flat.pdf

Life sciences study design

All studies must disclose on these points even when the disclosure is negative.

Sample size	4,268 available genomes from 139 different Bacillus species were downloaded from the NCBI database for phylogenetic tree construction and biosynthetic gene clusters (BGCs) prediction, and 545 representative Bacillus genomes were further selected for BGC distance calculation based on the following criteria: (i) high genome sequencing quality that available for further BGC distance calculation; (ii) covering all Bacillus species; (iii) for each species, the representative genome(s) was/were chosen from each of the main branches in the phylogram of genomes within this species.<br>For the colony confrontation assay, the antagonistic bacteria included eight heterogenic strains from the subtilis or cereus clade (two from subtilis subclade, two from pumilus subclade, and four from cereus clade), which are the two dominant groups within the genus Bacillus and both possess abundant secondary metabolites; the target bacteria included 59 strains covering all four phylogenetic clades, and their species are located on the main branches of the Bacillus tree. For the fermentation supernatant inhibition assay, the antagonistic and target bacteria included 17 and 40 strains, respectively, both of which cover all four clades and the relevant species are located on the main branches of the Bacillus tree. Both the colony confrontation and fermentation supernatant inhibition assay included three biological replicates for each interaction.<br>For the mutant-antagonism assay, the 11 antagonistic strains included wild-type Bacillus velezensis SQR9 and its 10 mutants that deficient in different BGCs or the sfp gene; the 24 target strains cover all four clades and the relevant species are located on the main branches of the Bacillus tree. All interactions included six biological replicates.<br>All these sample sizes are reflected in the Methods section.
Data exclusions	In the antagonism assay, several target strains grown abnormally as the incubation temperature (22℃) was much lower than their optimum growth temperature, therefore these data was not applicable.
Replication	4,268 available genomes from 139 different Bacillus species were downloaded for phylogenetic tree construction and biosynthetic gene clusters (BGCs) prediction, and 545 representative Bacillus genomes with high sequencing quality were further selected for BGC distance calculation. For colony confrontation assay, we selected eight antagonistic strains and 59 target strains; for fermentation supernatant inhibition assay, we selected 17 antagonistic strains and 40 target strains; all interactions included three biological replicates, respectively. For antagonism assay using Bacillus velezensis SQR9 and its mutants, we used 11 antagonistic strains including wild-type SQR9 and 10 constructed BGC-deficient mutants, and 24 target strains; all interactions included six biological replicates. Sample size is reflected in the Methods section.<br>All attempts at experiments replications were successful, except that several target strains in the antagonism assay grown abnormally as the incubation temperature (22℃) was much lower than their optimum growth temperature, and these data was not applicable.
Randomization	Randomization was not relevant in this study. Comparisons were not performed on individual organisms (with high level of individual variability) but on large population of bacteria cells that were independently initiated from small number of cells. Growth conditions were strictly controlled by the experimenter and identical for each population.
Blinding	Blinding was not relevant in this study, because methods used in the assessment of the results were objective. Blinding is also not necessary because the results are quantitative and did not require subjective judgment or interpretation. Blinding is not typically used in the field.

Reporting for specific materials, systems and methods

We require information from authors about some types of materials, experimental systems and methods used in many studies. Here, indicate whether each material, system or method listed is relevant to your study. If you are not sure if a list item applies to your research, read the appropriate section before selecting a response.

Materials & experimental systems

n/a	Involved in the study
☒	☐ Antibodies
☒	☐ Eukaryotic cell lines
☒	☐ Palaeontology and archaeology
☒	☐ Animals and other organisms
☒	☐ Human research participants
☒	☐ Clinical data
☒	☐ Dual use research of concern

Methods

n/a	Involved in the study
☒	☐ ChIP-seq
☒	☐ Flow cytometry
☒	☐ MRI-based neuroimaging
